# Relationship between 3p deletions and telomerase activity in non-small-cell lung cancer: prognostic implications

**DOI:** 10.1038/sj.bjc.6601775

**Published:** 2004-04-06

**Authors:** P Iniesta, R González-Quevedo, A Morán, C García-Aranda, C de Juan, A Sánchez-Pernaute, A Torres, E Díaz-Rubio, J L Balibrea, M Benito

**Affiliations:** 1 Departamento de Bioquímica y Biología Molecular, Facultad de Farmacia, Universidad Complutense, Madrid 28040, Spain; 2 Servicio de Cirugía Hospital Clínico San Carlos, Madrid 28040, Spain; 3 Servicio de Oncología, Hospital Clínico San Carlos, Madrid 28040, Spain

**Keywords:** chromosome 3p, deletions, telomerase activity, non-small-cell lung cancer, patient prognosis, tumorigenesis

## Abstract

3p deletions and telomerase reactivation are two of the most frequent events described in relation to non-small-cell lung cancer (NSCLC) pathogenesis. Moreover, a number of genes that map on 3p have been proposed as candidates to tumour-suppressor genes of importance in the lung cancer process. In this work, we analysed deletions at different 3p loci in relationship to telomerase activity in 66 NSCLCs obtained from patients who had suffered potentially curative surgery. Also, we evaluated prognostic implications. DNA samples were analysed for 3p deletions using five different polymorphic human dinucleotide repeat DNA markers (D3S1619 at 3p22.2, D3S3623 at 3p22.1, D3S1260 at 3p21.33, D3S3697 at 3p14.3, and D3S3722 at 3p21.2). Telomerase activity was investigated by a TRAP-based method. Possible correlations between the different molecular markers and distributions of disease-free survival were estimated. Our data revealed a significant correlation between telomerase activity and losses of heterozygosity (LOH) on D3S3697 (*P*=0.040), since all of the tumours showing deletion at this locus were positives for telomerase. Moreover, our results revealed clear associations with poor prognosis of patients, in the case of LOH at D3S1260 and D3S3697 (*P*=0.005 and 0.005, respectively). According to our data, potential repressors for telomerase may be located in chromosome 3p.

Lung cancer is one of the most lethal types of cancer to acquire, as reflected in a 5-year survival rate of only 14%. The high mortality rate for lung cancer results, at least in part, from the absence of standard clinical procedures for diagnosis of the disease at early and more treatable stages compared to other cancers. Molecular genetic studies have shown that mutations in proto-oncogenes and tumour-suppressor genes (TSGs) are critical in the multi-step development and progression of lung tumours. Inactivation of TSGs is by far the most common mutational event documented during lung tumorigenesis (Wiest *et al*, 1997). In this context, losses of heterozygosity (LOHs) on the short arm of chromosome 3 have been frequently reported in tumours from different origin, including lung tumours. In fact, allele loss involving chromosome 3p has been described as one of the most frequent and earliest known genetic events in lung cancer pathogenesis and it is thought that this may affect several potential TSG regions (Kok *et al*, 1997; Wistuba *et al*, 2000.

A number of genes that map on 3p have been proposed as candidates to TSGs of importance in the lung cancer process. One candidate is *FHIT* at 3p14.2, which undergoes frequent hemizygous and occasional homozygous deletion in lung cancer cells and encodes a dinucleoside hydrolase (Sozzi *et al*, 1996). Moreover, the reduced expression of the DNA mismatch repair gene *hMLH1* (3p21) has been correlated with allelic imbalance on chromosome 3p in non-small-cell lung carcinomas, and an association between *hMLH1* reduced expression and nodal metastasis in squamous cell carcinoma of the lung has been observed (Xinarianos *et al*, 2000). In addition, the 3p21.3 region has also been extensively examined for putative TSGs, particularly at a 600-kb region homozygously deleted in SCLC cell lines (Wei *et al*, 1996). More recently, a novel gene encoding a 1755-amino-acid polypeptide has been isolated at 3p22-21.3 and aberrant transcription of this gene, designated *DLEC1* (deleted in lung and oesophagus cancer 1), may be involved in carcinogenesis of the lung (Daigo *et al*, 1999).

In the last few years, acquired loss of the entire or parts of the short arm of chromosome 3 has been considered, by a few authors, in relationship to the lack of regulation of telomerase (Ohmura *et al*, 1995; Horikawa *et al*, 1998). In renal cell carcinomas, the presence of at least two genes with regulatory function on the expression of telomerase has been suggested (Mehle *et al*, 1998). Also, deletion analysis of nonrepressed segregant monochromosome 3 hybrids, in human breast cancer cells, indicated two regions on 3p (3p21.3–p22 and 3p12–21.1), where telomerase regulator genes may be located (Cuthbert *et al*, 1999).

Considering that 3p deletions and telomerase reactivation are two of the most frequent alterations described in relation to lung carcinogenesis, and that there is no report examining both alterations in non-small-cell lung cancer (NSCLC), in this work we have analysed LOH at different 3p loci in relationship to telomerase activity in 66 non-small-cell lung carcinomas obtained from patients who had suffered potentially curative surgery. Also, we have evaluated the prognostic implications for both molecular markers.

## MATERIALS AND METHODS

### Patients and tumour samples

In all, 66 freshly resected lung carcinoma samples were obtained from 66 patients who underwent surgery between 1995 and 2000 at the San Carlos Hospital in Madrid. This study was approved by the Ethical Committee from the Hospital, and informed consent from patients was obtained prior to investigation. Of the 66 patients, four were female and 62 were male, with an average age of 63.53±9.39 years. The median follow-up period for patients was 24 months (range 3–61 months). After surgical resection, samples were immediately frozen and were kept at −80°C until used. Molecular analyses were performed in samples containing >80% tumour cells. In all cases, nontumour tissues selected from macroscopically normal areas of surgical specimens were used as controls. Tumours were staged pathologically using the tumour node metastasis (TNM) system (Mountain, 1986) and consisted of 26 TNM Stage I tumours, six TNM II tumours, 26 IIIA tumours, seven TNM IIIB tumours, and one TNM Stage IV tumour. Therefore, 58 patients, who had stage I, II or IIIA tumours, were subjected to curative surgery, whereas only a biopsy was taken from patients who suffered from more extensive disease. According to the World Health Organisation criteria, 39 tumours were squamous cell carcinomas (SCC); 26 were adenocarcinomas (AC); and one was large-cell undifferentiated carcinoma (LCUC). The histological classification of tumours was established according to previous criteria (Sobin, 1982). Thus, 17 tumours were well differentiated; 31 moderately; and 18 poorly differentiated.

### Analysis of 3p LOHs

**Figure 1:**
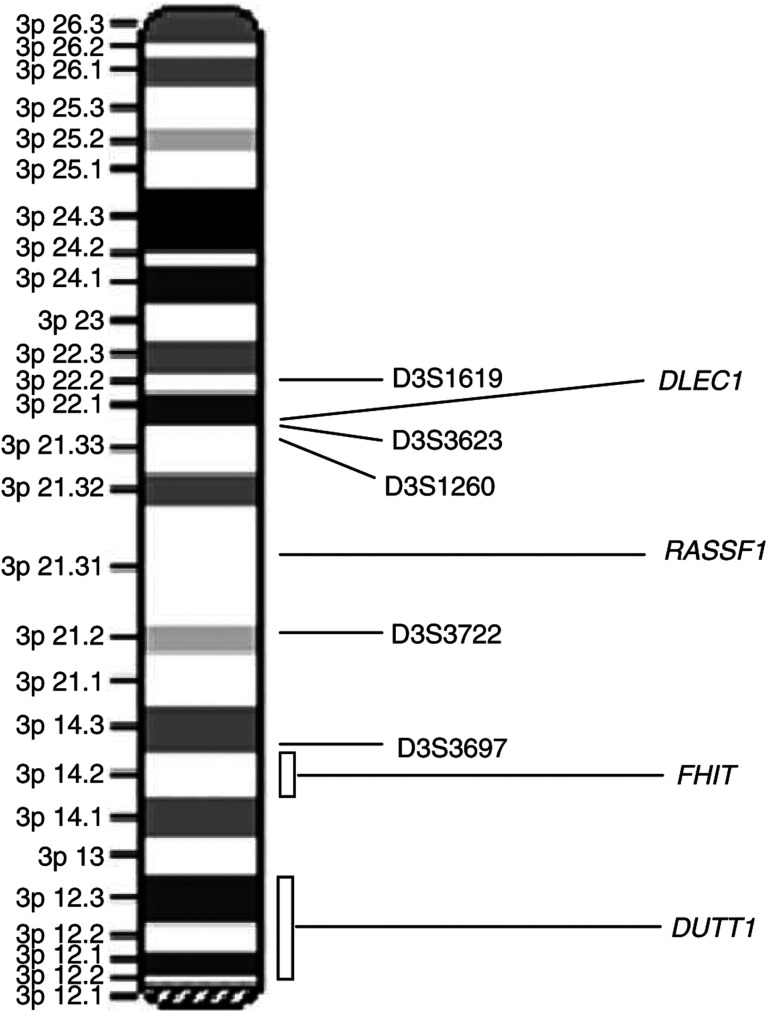
Diagram of chromosome 3p showing the microsatellite markers used in the LOH analysis. Genetic markers are listed in descending order from telomere to centromere on its approximate citogenetic (ideogram) positions. The positions of the *DLEC1*, *RASSF1*, *FHIT*, and *DUTT1* genes are also shown.

Genomic DNA was isolated from tumour and nontumour tissues as described previously (Blin and Stafford, 1976). DNA samples were amplified using polymerase chain reaction (PCR) and analysed for LOH on chromosome 3p using the five different polymorphic human dinucleotide repeat DNA markers (D3S1619 at 3p22.2, D3S3623 at 3p22.1, D3S1260 at 3p21.33, D3S3697 at 3p14.3, and D3S3722 at 3p21.2) previously reported (Cuthbert *et al*, 1999). Sequences for these markers were obtained from the National Centre for Biotechnology Information (NCBI) molecular databases. The relative positions of DNA microsatellite markers on 3p are indicated in Figure 1

. PCR was performed in a Thermocycler (Gene Amp PCR System 2400; Perkin-Elmer, Norwalk, CT, USA) and was carried out in a 20 *μ*l volume containing 100 ng of genomic DNA; 0.5 *μ*M of upstream and downstream primers; 0.2 mM of each deoxynucleoside triphosphate; 10 mM Tris-HCl, pH 8.3; 50 mM KCl; 2.5 mM MgCl_2_; and 0.5 U of Taq Gold DNA polymerase (Ecogen, SRL, Spain). PCR conditions were as follow: initial denaturation at 95°C for 10 min; 35 cycles at 95°C for 20 s, at 55°C for 20 s, and at 72°C for 30 s. The final extension was at 72°C for 7 min. After amplification, PCR products were resolved on an ABI 377 Sequencer and analysed by Genescan software (Applied Biosystems, USA). For informative cases, allelic loss was scored if one allele was significantly decreased in tumour DNA (>50%) compared to the same allele in normal control DNA.

### Evaluation of telomerase activity

Telomerase activity in paired normal and tumour tissues was evaluated as previously published (González-Quevedo *et al*, 2000), using a Telomerase PCR ELISA Kit from Roche (Roche Molecular Biochemicals, Germany). The method is an extension of the original Telomeric Repeat Amplification Protocol (TRAP) (Kim *et al*, 1994). Briefly, tissue samples were lysed in ice-cold buffer for 30 min. In the first step, a volume of cell extract containing 6 *μ*g of total proteins was incubated with a biotin-labelled synthetic telomerase-specific primer, and under established conditions the telomerase present in cellular extracts adds telomeric repeats (TTAGGG) to the 3′ end of the primer. Next, in the second step, these elongation products were amplified by PCR using specific primers. An aliquot of the PCR products was denatured, hybridised to a digoxigenin-labelled, telomeric repeat-specific probe, and bound to a streptavidin-coated microtitre plate. The immobilised PCR products were then detected with an antibody against digoxigenin that is conjugated to peroxidase (anti-DIG-POD). Finally, the probe was visualised by virtue of peroxidase-metabolising TMB to form a coloured reaction product, and semiquantified photometrically (450 nm). Considering that the cutoff for TRAP-ELISA negativity corresponds to OD_450 nm_ <0.2, all samples showing OD_450 nm_ >0.2 were judged as telomerase activity positive. As positive control, we used an extract of the telomerase embryonic kidney cell line 293, and negative controls were prepared in each case by treating cell extracts with RNase (DNase-free). Moreover, to avoid the effect of Taq polymerase inhibitors present in the tissue extracts, we estimated the activity of telomerase by serial dilutions of each extract as described previously (Kim *et al*, 1994).

### Statistical analysis

Both 3p deletions and telomerase activity were assessed for potential associations with a number of clinicopathologic parameters, including patient gender and age, and TNM stage, histology and differentiation grade of the primary tumours. Moreover, possible associations between the telomerase activity of tumours and LOHs at the different markers considered were evaluated. The relationship between categorical variables was assessed using the χ^2^ test. A *P-*value <0.05 was judged to be significant. Distributions of disease-free survival (DFS) were estimated with the Kaplan–Meier method, and comparisons were made with log-rank statistics. Results were considered significant for *P*-values <0.05. For survival analysis, only patients who had undergone potentially curative surgery (patients with TNM stages I–IIIA tumours) were considered. Thus, the number of patients included in the survival study was 58. Analysis was performed using windows SPSS version 11.0 software.

## RESULTS

Loss of heterozygosity analyses were performed in 66 cases of NSCLC. In addition, the normal bronchial tissue from each case was tested simultaneously as a control. Five microsatellite markers mapping on chromosome 3p were chosen to evaluate LOH in the DNAs from all of the samples described in Materials and Methods section. According to our data, the heterozygosity rate for DNA markers tested here was an average of 80% or greater.

Allelic losses on 3p were recorded in 66.7% (44 out of 66) of tumours. Results for individual markers revealed that D3S1619 represented the most frequently altered locus since, only considering informative cases, 20 of 61 tumours (32.7%) showed LOH at this chromosome locus. Loss of heterozygosity frequency for the other markers investigated here revealed that 20.7, 20, 24.2, and 24.2% of informative cases showed deletions at D3S3623, D3S1260, D3S3697, and D3S3722, respectively. In relation with clinico-pathological variables, we did not find any significant correlation with the 3p deletions investigated in this work (data not shown).

Telomerase activity was positive in 55 (83.3%) of 66 NSCLC tissue specimens analysed and negative in 11 (16.7%) cases. The relationship between telomerase activity and clinical and pathologic features is summarised in Table 1

**Table 1:** Telomerase activity and clinico-pathological variables

		**Telomerase activity**		
**Variable**	**No of cases**	**(+) (%)**	**(−) (%)**	***P*-value**
Age	66	63.78±9.32	62.27±10.12	0.630
				
*Gender*				
Males	62	53 (85.4)	9 (14.6)	0.126
Females	4	2 (50)	2 (50)	
				
*Tumour stage*				
I	26	20 (76.9)	6 (23.1)	0.526
II	6	5 (83.3)	1 (16.7)	
IIIA	26	24 (92.3)	2 (7.7)	
IIIB	7	5 (71.4)	2 (28.6)	
IV	1	1 (100)	(0)	
				
*Histology*^a^				
SCC	39	32 (82)	7 (18)	0.871
AC	26	22 (84.6)	4 (15.4)	
LCUC	1	1 (100)	0 (0)	
				
*Differentiation*				
Well	17	11 (64.7)	6 (35.3)	0.018
Moderate	31	26 (83.8)	5 (16.2)	
Poor	18	18 (100)	0 (0)	

a SCC, squamous cell carcinoma; AC, adenocarcinoma; LCUC, large-cell undifferentiated carcinoma.

. The mean ages at surgery, sex, tumour stage, and histology were not different between the two groups. However, telomerase activity was significantly correlated with poor differentiation of cancers (Table 1).

When we studied the possible associations between LOHs on 3p and telomerase reactivation, our data revealed a significant correlation with LOH on D3S3697 (3p14.3) (*P*=0.040). In fact, all of the tumours showing LOH at this locus were positives for telomerase activity (Table 2

**Table 2:** Relationship between telomerase activity and losses of heterozygosity on the different markers considered

		**Telomerase activity**		
**Locus**	**No of cases**	**(+) (%)**	**(−) (%)**	***P*-value**
*D3S1619*				
LOH (+)	20	14 (70)	6 (30)	0.092
LOH (−)	41	36 (87.8)	5 (12.2)	
				
*D3S3623*				
LOH (+)	11	7 (63.6)	4 (36.4)	0.112
LOH (−)	42	36 (85.7)	6 (14.3)	
				
*D3S1260*				
LOH (+)	12	12 (100)	0 (0)	0.087
LOH (−)	48	38 (79.2)	10 (20.8)	
				
*D3S3697*				
LOH (+)	16	16 (100)	0 (0)	0.040
LOH (−)	36	28 (77.8)	8 (22.2)	
				
*D3S3722*				
LOH (+)	16	12 (75)	4 (25)	0.401
LOH (−)	39	32 (82.1)	7 (17.9)	

). As it can be also observed in Table 2, this analysis indicated a borderline association between D3S1619 (3p22.2) or D3S1260 (3p21.33) deletions and positivity for telomerase. Moreover, 100% of samples showing LOH at D3S1260 had been classified in the group of telomerase-positive cases.

Next, we performed a survival analysis for each one of the variables evaluated in this work. Thus, such as it had been previously reported (González-Quevedo *et al*, 2002), the Kaplan and Meier survival curves for patients with NSCLC demonstrated that patients with telomerase-positive tumours survived for a shorter period than those with telomerase-negative cancers (*P*=0.04).

**Figure 2:**
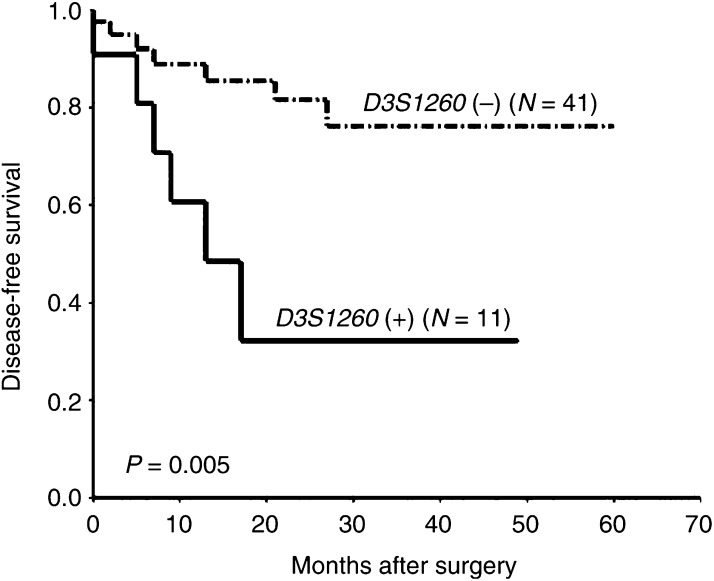
Correlation between LOH at D3S1260 and DFS in patients with NSCLC. N: number of cases; (−): LOH negative; (+): LOH positive.

In relation to 3p deletions, survival studies indicated a diminished DFS time in patients affected by tumours showing this molecular abnormality, but differences were not statistically significant (data not shown). Interestingly, when survival impact was evaluated individually for each one of the DNA markers, only considering informative cases, our results revealed clear associations with poor prognosis of patients in the case of LOH at D3S1260 (3p21.33) and D3S3697 (3p14.3) (*P*=0.005 and 0.005, respectively) (Figures 2

and 3

**Figure 3:**
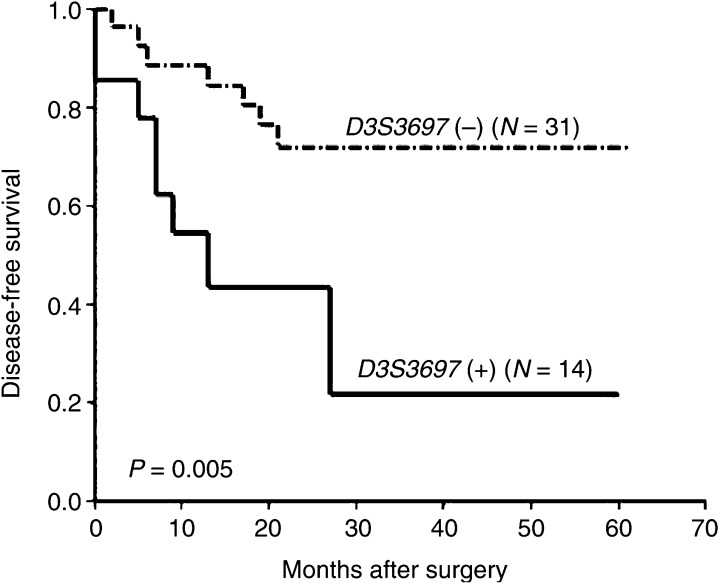
Kaplan–Meier survival curves for LOH at D3S3697 in NSCLC patients. N: number of cases; (−): LOH negative; (+): LOH positive.

).

## DISCUSSION

Deregulation of telomerase expression has been directly linked to human diseases, including cancer. The expression of telomerase is suppressed in most normal human somatic cells, but is reactivated during tumorigenesis. This reactivation seems to arrest the normal loss of telomeric DNA incurred as somatic cells divide. Since continual loss of telomeric DNA is predicted to eventually limit cell proliferation, activation of telomerase in cancer cells may be an important step in the acquisition of cell immortalisation, which occurs during tumour progression (Counter *et al*, 1998; Meyerson, 2000). The mechanisms involved in telomerase regulation are still far from being fully established (Cong *et al*, 2002). Thus, identification of transcriptional repressors and tumour-specific activators of telomerase subunits should have considerable impact on the understanding of cellular senescence and immortalisation.

Non-small-cell lung cancer represents one of the most frequent fatal malignancies in the world. Chromosome 3p deletions have been described as one of the most frequent genetic events in lung cancer pathogenesis and it is though that may affect several potential TSG regions (Kok *et al*, 1997; Wistuba *et al*, 2000).

During the last few years, several authors have reported potential associations between telomerase reactivation and 3p deletions. Thus, restoration of the cellular senescence program by chromosome 3 has been associated with repression of telomerase function in renal cell carcinoma (Ohmura *et al*, 1995). Moreover, it has been suggested that a senescence-inducing gene on chromosome 3 controls *hEST2/hTERT* gene expression either directly or indirectly, and support the notion that hEST2/hTERT is the major determinant of telomerase enzymatic activity in human cells (Horikawa *et al*, 1998). More recently, a strong repression of telomerase was observed following transfer of human chromosome 3 into human breast cancer cells (Cuthbert *et al*, 1999). Authors of this work identified two regions on the short arm of chromosome 3 (3p21.3-p22 and 3p12-21.1) where telomerase regulator genes may be located.

Considering the importance of telomerase reactivation and 3p deletions in NSCLC pathogenesis and, on the basis of previous reports, we have investigated possible associations between both molecular markers in non-small-cell lung tumours obtained by surgery from patients affected by this pathology. Losses of heterozygosity on 3p have been evaluated analysing microsatellite markers that map on the regions previously reported as important for repressing telomerase (Cuthbert *et al*, 1999). The validity of the five DNA markers used here for 3p deletion investigation was corroborated. Thus, our data indicated that the heterozygosity rate for these microsatellite markers was an average of 80% or greater in all cases.

Almost 70% of the tested samples showed 3p deletion in at least one of the loci considered. In spite of these results agreeing with the data previously reported by other authors (Mitsudomi *et al*, 1996), other studies show higher rates of genetic losses in the chromosome 3p region in lung cancer. Thus, Wistuba *et al* (2000) detected one or more regions of 3p allele loss in nearly all lung cancer cell lines and in 97% of all resected primary lung tumours. According to these authors, 3p deletions progress in frequency and in size with increasing severity of histopathological changes. The differences in 3p LOH incidences could be attributed to the histological characteristics of tumours. Thus, differences in the allelic loss and mutation patterns have been reported previously between squamous and adenocarcinomas, suggesting that more genetic changes accumulate during tumorigenesis in squamous cell carcinomas than in adenocarcinomas (Sato *et al*, 1994). This fact may be related to smoking damage (Gazdar and Minna, 1997). However, no association between 3p LOH and smoking exposure was detected in our resected lung carcinoma cases.

On the other hand, our results for telomerase indicated that 83.3% of tumours showed positive activity. These incidences are also similar to that obtained in other previous works (Taga *et al*, 1999). Furthermore, according to our results, this molecular parameter represented a poor prognosis indicator in NSCLC. In fact, all recurrences were detected in the group of patients with positive telomerase tumours.

When the relationship between telomerase reactivation and 3p deletions at the different loci considered in the present study was established, results obtained showed a significant correlation between telomerase activity and LOH at D3S3697 (mapping at 3p14.3). Moreover, borderline associations were found for LOH at D3S1619 or D3S1260 (located at 3p22.2 and 3p21.33, respectively) and telomerase-positive cases. Interestingly, LOH on two of the three DNA markers was present only in telomerase-positive samples (D3S3697 and D3S1260) and, such as it can be observed in the Results section, survival studies demonstrated the importance of LOHs at these levels in poorer patient prognosis.

Therefore, according to our results, the potential repressors for telomerase may be located in chromosome 3p, and to our knowledge this is the first work in which such association is investigated ‘*in vivo*’ in NSCLC tumours. Although the nature of 3p genes implicated in negative regulation of telomerase remains unknown, according to our results, possible TSGs may be involved as candidates. In fact, several genes with importance in lung development and bronchial hyperplasia are located near to the chromosome region where D3S3697 maps (3p14.3) (Figure 1). Between these, two suppressor genes have been proposed. Thus, *FHIT* (at 3p14.2), which undergoes frequent deletion in lung cancer cells and encodes a dinucleoside hydrolase (Sozzi *et al*, 1996), could be affected by LOH at D3S3697. On the other hand, *DUTT1* (3p12–13), coding for a receptor with a domain structure of the neural-cell adhesion family, could also be considered. This gene is widely expressed and has been implicated in the guidance and migration of axons myoblasts, and leukocytes in vertebrates. Mice experiments demonstrated that deletion of the 3p region where *DUTT1* maps was associated with bronchial epithelial abnormalities including hyperplasia (Xian *et al*, 2001).

Moreover, a novel gene has been mapped in 3p22–21.3 (Figure 1). This gene, called *DLEC1* (deleted in lung and oesophagus cancer 1) (Daigo *et al*, 1999) has been identified as a candidate TSG, and it has been suggested that it may be involved in carcinogenesis of the lung, oesophagus, and kidney. Our results suggest that deletions in D3S1619 or D3S1260 could be related to LOHs affecting this gene.

Finally, in the group of TSGs on 3p that may be considered as candidates to negative regulation of telomerase, *RASSF1* would be included (Figure 1). This putative tumour suppressor identified at 3p21.3 has recently been analysed in relation to NSCLC. Silencing of this gene occurs mainly by promoter methylation (Endoh *et al*, 2003; Kim *et al*, 2003), but gene deletion has also been described as a potential mechanism that contributes to lung tumorigenesis (Kuroki *et al*, 2003).

Therefore, results included in this work corroborate ‘*in vivo*’ previous data obtained ‘*in vitro*’, suggesting the existence on 3p of one or several genes that participate in telomerase-negative regulation. However, according to gene databases, a wide group of genes on 3p could be related to telomerase regulation, and future and exhaustive studies examining deletions affecting different regions of each one of these genes in relation to telomerase activity are necessary in order to elucidate mechanisms involved in telomerase regulation. In fact, identification of telomerase repressors should have considerable impact on the understanding of cellular immortalisation and cellular senescence, as well as on cancer therapeutic based on the use of telomerase inhibitors.
